# P-365. Pooled safety and tolerability of twice-yearly lenacapavir with teropavimab and zinlirvimab for HIV-1 treatment

**DOI:** 10.1093/ofid/ofaf695.583

**Published:** 2026-01-11

**Authors:** Kimberly Workowski, Peter Ruane, Anthony Mills, Mehri S McKellar, Olayemi O Osiyemi, Linda Gorgos, Gordon E Crofoot, Moti Ramgopal, Cynthia C Brinson, Kwad Mponponsuo, Sean E Collins, Hui Liu, Nan Zhang, Keith Flower, Tina Chakrabarti, Onyema Ogbuagu

**Affiliations:** Department of Medicine, Emory University, Atlanta, GA, USA, Atlanta, Georgia; Ruane Clinical Research, Los Angeles, CA, USA, Los Angeles, California; Men's Health Foundation, Los Angeles, CA, USA, Los Angeles, California; Division of Infectious Diseases, Department of Medicine, Duke University School of Medicine, Durham, NC, USA, Durham, NC; Triple O Research Institute PA, West Palm Beach, Florida, USA, West Palm Beach, Florida; AXCES Research Group, Santa Fe, NM, USA, Santa Fe, New Mexico; The Crofoot Research Center, Houston, TX, USA, Houston, Texas; Midway Immunology and Research Center, Fort Pierce, Florida; Central Texas Clinical Research, Austin, TX, USA, Austin, Texas; Gilead Sciences, Inc., Foster City, CA, USA, Foster City, California; Gilead Sciences, Inc., Foster City, CA, USA, Foster City, California; Gilead Sciences, Inc., Foster City, CA, USA, Foster City, California; Gilead Sciences, Inc., Foster City, CA, USA, Foster City, California; Gilead Sciences, Inc., Foster City, CA, USA, Foster City, California; Gilead Sciences, Inc., Foster City, CA, USA, Foster City, California; Yale School of Medicine, New Haven, CT, USA, New Haven, Connecticut

## Abstract

**Background:**

Lenacapavir (LEN), the HIV-1 capsid inhibitor, combined with the broadly neutralizing antibodies teropavimab (TAB, GS-5423) and zinlirvimab (ZAB, GS-2872), is an investigational twice-yearly (Q6M) regimen for HIV-1 treatment. In Phase 1b (NCT04811040) and Phase 2 (NCT05729568) studies, LEN, TAB, and ZAB maintained virologic suppression (HIV-1 RNA < 50 copies/mL) for 6 months after one dose. We evaluated pooled Phase 1b/2 safety and tolerability of LEN, TAB, and ZAB, including the effects of anti-drug antibodies (ADAs) against TAB and ZAB on safety.

**Table 1.**
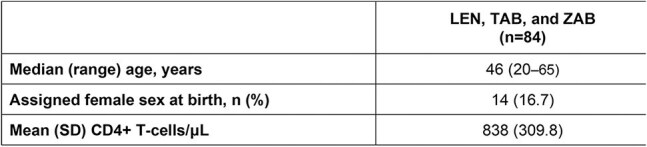
Pooled Baseline Characteristics LEN, lenacapavir; TAB, teropavimab; ZAB, zinlirvimab.

**Table 2.**
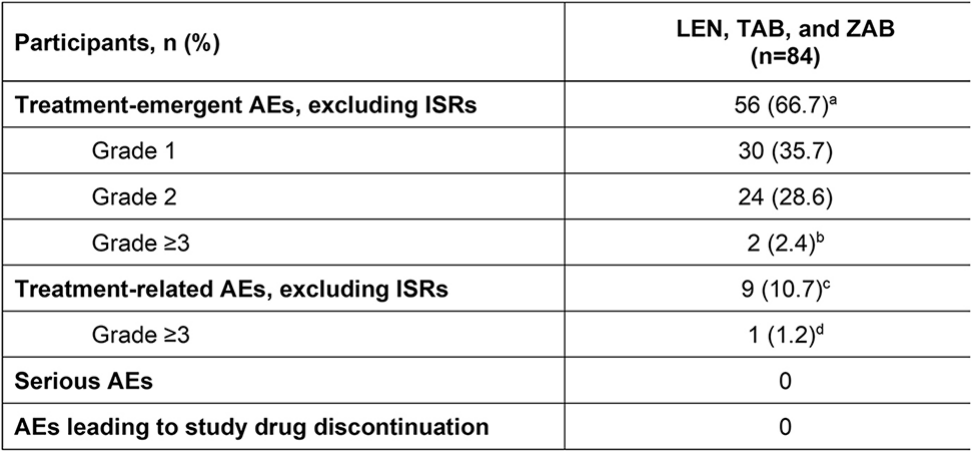
Pooled Safety Outcomes at Week 26 a.Including ISRs, 71 participants (84.5%). b.Acute pyelonephritis, ureteritis, and nephrolithiasis in 1 participant; injection site cellulitis in 1 participant (not considered an ISR per Medical Dictionary for Regulatory Activities 26.1). c.Increased lacrimation, diarrhea, headache, nausea, abnormal dreams, insomnia, device dislocation, infusion-related reaction, injection site cellulitis (n=1 each). Including ISRs, 57 participants (67.9%). d.Injection site cellulitis. AE, adverse event; ISR, injection site reaction; LEN, lenacapavir; TAB, teropavimab; ZAB, zinlirvimab.

**Methods:**

Both studies enrolled virologically suppressed adults with HIV-1. In Phase 1b, participants were randomized 1:1 across two cohorts to receive one dose of 927 mg subcutaneous (SC) LEN (+ oral loading), 30 mg/kg intravenous (IV) TAB, and either 10 mg/kg or 30 mg/kg IV ZAB. In Phase 2, participants were randomized 2:1 to switch to Q6M 927 mg SC LEN (+ oral loading), 2550 mg IV TAB and 2550 mg IV ZAB, or to continue daily oral stable baseline regimen (SBR) through Week (W)52. Safety and ADA data through W26 for those receiving LEN, TAB, and ZAB were pooled.

**Results:**

The pooled analysis included 84 participants (Phase 1b, n=31; Phase 2, n=53); baseline characteristics are shown in Table 1. Excluding injection site reactions (ISRs), treatment-emergent adverse events (AEs) occurred in 56 (66.7%) participants (Table 2). ISRs related to SC LEN were reported in 55 (65.5%) participants and in most (47/55) these were Grade 1 (mild). There were no infusion-related reactions, serious AEs, or AEs leading to study drug or study discontinuation. Grade ≥3 laboratory abnormalities occurred in 5 (6.0%) participants and were judged not clinically significant by the investigator. Safety profiles for the pooled LEN, TAB, and ZAB cohort were comparable to the Phase 2 SBR cohort. ADAs against TAB were detected in 11 (13.3%) participants and against ZAB in 15 (18.1%). ADAs were generally observed at low titers and did not have an identifiable effect on safety.

**Conclusion:**

LEN, TAB, and ZAB were well tolerated with a favorable safety profile up to W26 across Phase 1b and Phase 2 studies. ADAs against TAB and ZAB were low in titer and did not impact safety. The ongoing Phase 2 study will continue to evaluate LEN, TAB, and ZAB as the first complete twice-yearly regimen for HIV-1 treatment.

**Disclosures:**

Kimberly Workowski, MD, Gilead Sciences, Inc.: Institutional research support Peter Ruane, MD, Gilead Sciences, Inc.: Advisor/Consultant|Merck: Advisor/Consultant|ViiV: Advisor/Consultant Olayemi O. Osiyemi, MD, Gilead Sciences, Inc.: Advisor/Consultant|Gilead Sciences, Inc.: Honoraria|Merck: Advisor/Consultant|Merck: Honoraria|ViiV: Advisor/Consultant|ViiV: Honoraria Linda Gorgos, MD, MSc, FIDSA, Akero Therapeutics: Grant/Research Support|Gilead Sciences, Inc.: Grant/Research Support|Glaxo SmithKline/ViiV: Grant/Research Support|Merck Sharp and Dohme: Grant/Research Support|Moderna: Grant/Research Support|Novavax: Grant/Research Support Gordon E. Crofoot, MD, Gilead Sciences, Inc.: Grant/Research Support Moti Ramgopal, MD, AbbVie: Honoraria|Gilead Sciences, Inc.: Advisor/Consultant|Gilead Sciences, Inc.: Honoraria|Shionogi: Advisor/Consultant|ViiV: Advisor/Consultant|ViiV: Honoraria Cynthia C. Brinson, MD, Gilead Sciences, Inc.: Grant/Research Support|Gilead Sciences, Inc.: Honoraria|Gilead Sciences, Inc.: Medical writing funds|GSK: Grant/Research Support|ViiV: Grant/Research Support Kwad Mponponsuo, MD, MSc, Gilead Sciences, Inc.: Employee and shareholder Sean E. Collins, MD, MS, Gilead Sciences, Inc: Employee Hui Liu, PhD, Gilead Sciences, Inc.: Employee|Gilead Sciences, Inc.: Stocks/Bonds (Private Company) Nan Zhang, PhD, Exelixis: Employee|Gilead science, inc.: Employee|Gilead science, inc.: Stocks/Bonds (Public Company) Keith Flower, MD, Gilead Sciences, Inc.: Employee|Gilead Sciences, Inc.: Stocks/Bonds (Public Company) Tina Chakrabarti, MD, Gilead Sciences, Inc.: Employee Onyema Ogbuagu, MA, FACP, FIDSA, Gilead Sciences, Inc.: Advisor/Consultant|ViiV: Advisor/Consultant

